# Simple ideas that work: Celebrating development in persons with profound intellectual and multiple disabilities

**DOI:** 10.4102/ajod.v7i0.273

**Published:** 2018-06-05

**Authors:** Ann Bullen, Rosemary Luger, Debbie Prudhomme, Martha Geiger

**Affiliations:** 1 The Chaeli Campaign, Cape Town, South Africa; 2 Centre for Rehabilitation Studies, Stellenbosch University, South Africa

## Abstract

**Background:**

The purpose of this article is to share some lessons learnt by an interdisciplinary therapy team working with persons with profound intellectual and multiple disabilities (PIMD), implemented in diverse, low-income contexts over a period of 8 years.

**Objectives:**

The objective of all the activities described here was to provide increased stimulation and development opportunities for persons with PIMD within different settings (day care centre, residential centre or family home).

**Method:**

We used an iterative action-learning approach where we applied existing evidence in the given context, reflected on and adapted strategies in collaboration with stakeholders on a cyclical basis. We focussed on achieving our objectives through ongoing hands-on training of the carers involved with the clients as we felt that by providing them with the knowledge and skills needed, plus ongoing support, these programmes would be more sustainable.

**Findings:**

It took some time to put systems in place in care settings, but once they became part of the daily routine, they provided increased opportunities for learning for clients with PIMD. In addition, there were often marked changes in individual clients’ communicative and physical functioning, which in turn encouraged carers to find new and different ways to interact with, and stimulate, the persons with PIMD in their care.

**Conclusion:**

Our hope is that parents and carers or professionals working in the field of PIMD in low-income contexts elsewhere may find one, some or all of these simple ideas useful in providing opportunities for learning, development and enjoyment for persons with PIMD.

## Purpose

The purpose of this article is to share some practical lessons learnt by an interdisciplinary team in the non-governmental sector, working with persons with profound intellectual and multiple disabilities (PIMD), in diverse contexts over a period of 8 years. Our team comprised a physiotherapist, an occupational therapist, a speech therapist and an educator. The settings included two residential and two day care centres catering for approximately 150 children and adults with PIMD in low-income contexts around Cape Town, South Africa.

Swartz (2014) has identified five interrelated challenges in implementing disability research in sub-Saharan Africa, namely experience, expertise, enumeration, evidence and expectations. Carefully negotiating these strategies, our long-term aim as a therapy team is to implement more rigorous research on effective, culturally appreciative and sustainable interventions for clients with PIMD in low-income contexts. In the meantime, we echo the value of sharing ‘what might be described as several of the many straightforward, practical schemes implemented … to achieve very specific goals within particular contexts’ (Zimmermann 2005:5). Our motivation for this article was a sharing of ideas that others working with clients with PIMD can try out and adapt rather than a scientific report.

## Background

The population of persons with PIMD is described as having a combination of cognitive impairment and neuromotor dysfunction (Nakken & Vlaskamp 2007). While South Africa has some of the most progressive human rights–based policies, the care and development of persons with PIMD remains an area not considered a priority with the available resources (Adnams 2010; McKenzie, McConkey & Adnams 2013b; Western Cape High Court 2011). This group of clients – and especially those in low-income contexts – along with their parents, carers and rehabilitation professionals face significant challenges (Aldersey 2012; Dambi, Jelsma & Mlambo 2015; McKenzie & McConkey 2016).

Institutional, residential care is not considered best practice – or even *good* practice – for children and adults with PIMD (Goozner 2013; The Presidency, RSA 2002; Wang et al. 2007). It is, however, a reality in South Africa, where some parents cannot cope owing to their employment needs and cannot find other home-based alternatives (Mckenzie, McConkey & Adnams 2013a); or where, in extreme cases, clients are removed from their families by the courts in the clients’ best interest. While efforts continue to re-integrate as many persons with PIMD in their families and communities as possible, those remaining in institutions have the right to best possible holistic care, stimulation and quality of life in that situation.

Similarly, while children in South Africa have a constitutional right to basic education, those with PIMD are excluded from even special schools and thus many attend day care centres for children with special needs where there is no formal curriculum and until recently very limited government support (Western Cape High Court 2011).

In most of the centres where members of The Chaeli Campaign Therapy Team have worked, the clients’ basic care needs (in terms of washing, feeding, changing and medication) were adequately – and caringly – met. However, most clients themselves were not actively engaged in these activities; moreover, the rest of the time at the day care or residential centre included little or no stimulation. This was partly because of limited human resources where the client to carer ratio was high, the staff did not have teacher training and there was little, if any, therapeutic support other than sporadic visits to individual, hospital-based therapy appointments.

Ethically, it is difficult in such circumstances to decide who should or should not get therapy input, and under such conditions we confirmed earlier observations that it was impractical to expect carers to implement individual therapy programmes (Geiger 2012; Van der Linde 2014).

Our goal, as reported in this article, was to find ways to improve the quality of life and functioning of each client with PIMD in a variety of areas, including basic interaction and communication, self-help where possible, sensory awareness and processing, positioning and mobility, social skills and cognition through practical capacity-building and empowerment of their carers.

### What we did and why

We adopted an action–learning approach (Adams 2010; Kemmis & McTaggart 2003; Trehan & Rigg 2012), which comprises cycles of action, observation, collaborative reflection and adjusted action. Three features were closely interrelated, that is, application of existing evidence in the given contexts, reflection and adaptation of strategies in collaboration with stakeholders and ‘iterancy’ (repeated cycles of planning, action, reflection and adjusted action).

We also applied an asset-based approach by using resources already available within the specific environment (Eloff & De Wet 2009) and a systems approach whereby over time we tried to enrich *systems* or the way routine, everyday things were done within the centre as a whole. This is in line with an ‘ecological’ systems approach, where the focus is upon the person’s environment and the learning opportunities created, rather than just on the person’s impairment (Guralnick 2011; Patel et al. 2008).

To enhance our input, we the authors, all members of The Chaeli Campaign therapy team, endeavoured to become an interdisciplinary team in terms of the definitive criterion of such a team as proposed by both Newell (2011) and Repko (2011), that is, integrating elements from diverse disciplines for the achievement of a specific goal. This involved interdisciplinary learning, role sharing, role acceptance and role release, which although initially challenging became easier with experience and as we built trusting relationships among ourselves.

In each of the centres supported, we added activities to the existing daily routine (Wilder & Granlund 2015) that could address the varying needs of the individual clients with the available human resources to complement what was already in place. We collaborated with the relevant facility’s management team to put in place systems that would help in facilitating growth of clients in the diverse areas we had identified. To support these systems, we spent weekly hands-on time in the centres with the carers and clients, role modelling therapeutic activities in group settings and facilitating problem-solving *in situ*. In addition, we facilitated quarterly workshops for staff using a participatory adult education approach (Werner & Bower 1982) and concentrated on building upon the skills they already had, taking into account the needs of the specific centre at the time, and accommodating changing needs over time.

Working in settings over a long period of time allowed us the chance to put in place creative systems to improve individual clients’ opportunities to learn and through this improve their functional abilities, as well as enhance their quality of life. Early on with the different centres there was a need to focus on standalone issues like the correct use of assistive devices for positioning and seating, feeding techniques and techniques to help residents cough and clear phlegm, but as these improved the training moved towards general stimulation, effective use of different experiential areas, communication, encouraging clients to become more independent in activities of daily living and using weekly themes to keep things varied and interesting for everyone. Finally, the training moved on to how programmes could be effectively monitored and evaluated with a simple checklist being used for self-evaluation by carers as well as evaluation of groups including correct seating by management staff (Appendix 1).

## Activities and outcomes

Nine examples of activities (each based upon specific, collaboratively identified needs, implemented, reflected upon and then adjusted) and their outcomes as per our observations as well as written and verbal feedback from staff are described below. The ‘simplicity’ of these ideas makes them easy to understand and implement elsewhere in a variety of settings.

### Small group stimulation time

In centre-based environments, this was usually introduced as a 1–2 h part of the morning routine after the clients’ basic care needs had been met. Clients were assigned to a carer according to their age or abilities in groups of six to eight. The small groups started in a circle with a greeting song, followed by a movement activity, art or constructive play, sensory stimulation such as massage, reading of a book or telling of a story and a song to finish off. Good positioning of each client on their own seating equipment was the starting point and as the carers became more actively involved in the groups and interested in trying out new activities, it became important to find ways for them to each have their own stimulation time box for which they were responsible.

#### Outcomes

From clients lying in bed, sitting alone on the floor or being placed in their buggies^1^ or wheelchairs in a straight line all facing the same direction, there evolved smaller groups in allocated areas facing each other. Initially, there was a lot of resistance to this stimulation group ‘adding to the load’ of the carers, plus they preferred to work with each other with larger groups of clients, but over time these small groups became a normal part of the daily routine. Carers displayed their own strengths in how they ran their groups and became creative with what activities or subjects they would introduce each day – new songs, objects from outside of the centre (nests, autumn leaves, pictures of their own families and even garden snails!). There was more communication between group members and friendships developed while previously communication was mainly between carers and clients and the carers themselves seemed more energised by this environment. Many were surprised by how much the clients in their group could understand and do when given opportunities.

### Toy library

Toys donated to centres are often not appropriate; therefore, initially time was spent sorting through donations and building up a library of appropriate toys for the specific needs of clients at the centres. The toys needed to be hardy and encourage more constructive play or be useful for sensory stimulation. There needed to be a system that ensured each carer including both day shifts in residential settings was able to access the toy library regularly. Support around choosing appropriate toys and activities for the individual group members was provided, especially when there was a change in their functional ability.

#### Outcomes

Although the toy library was initially time-consuming and carers felt little responsibility towards the items, this changed over time. Every carer having their own stimulation box or cupboard helped this. The carers became more actively involved in making choices about what to select and were keen to discuss with the occupational therapist in the therapy team what changes were happening with individual group members, likes or dislikes as well as how one could progress the use of items when there were functional improvements. Even though there was normal wear and tear of items chosen for the boxes, less of these were misplaced or lost as time went on. Over time, it became possible for our occupational therapist to slowly withdraw direct support and hand over this role to a member of the management team. The management teams also learnt which toy donations to include in their wish lists for donors. In the centres where we were involved, it seemed that having the toy library once a quarter was the most sustainable.

In another development, different *experiential areas* were introduced to increase the variety of experiences for clients at the different centres and to give them the opportunity to move away from their usual environment. So, we created spaces for a simple sensory area, a play area and an obstacle course (Box 1).

Box 1 Sensory area routine.

1. Turn on one of the lamps and ask clients to look at the light coming through the window and the glow-in-the dark shapes on the walls.
2. Ask everyone to listen – highlight any sounds in the environment, for example, birds, wind, people, etc.
3. Turn on the CD player with one of the CDs in and play it softly.
4. Massage everyone’s hands or feet using the perfumed cream.
5. Give clients something to hold in their hands and/or smell, for example, lavender beanbag, soft toy, shell, pinecone or aroma dough.
6. Tell clients that it is the end of the session.
7. Turn off electrical equipment.

*Source*: Authors’ own work

### Sensory area

Although we realised that having a group session was not ideal, we felt that exposing a small group of clients to a variety of sensory experiences would also be valuable. Carers with their clients used the routine in Box 1 to stimulate the different senses in a designated room where possible or in a quiet corner that could be screened off.

#### Outcomes

In the beginning, the logistics of a carer taking 6–8 clients (some in buggies or wheelchairs) to a sensory area was a logistical challenge. Carers were able to help one another move clients to the sensory area and clear guidelines with photos were also provided to remind carers of the routine, thereby contributing to the sustainability of the programme. In some centres, a timetable needed to be drawn up for sensory room visits. We had initially introduced this to give a sensory experience to the more physically disabled residents who were unable to access other forms of stimulation, but it also turned out to be very useful for the more physically able clients with challenging behaviour. Initially it was used as a calming space to manage behaviour when individual clients were overwrought but over time it became a reward for good behaviour.

### Themed room

There was a need for some of the higher functioning clients to have more intellectual stimulation than was provided by the daily small group stimulation time. To increase the learning opportunities for these clients, we set up areas to participate in facilitated themed activities, for example relating to phones, babies, animals, cars and occupations (Lifter et al. 2011). A routine was again created for carers to use with their small groups starting with a song related to the theme. They then passed relevant items around the group, discussed the theme, for example What sounds?/How do we use it?/Look after it?/What does it need?/How many?/What colour? Carers then told or read a story related to the day’s theme; there was a time for free play time where the group member could indicate what toy they would like to play with and the groups finished with a repeat of the initial song.

#### Outcomes

Initially, the carers were quite reserved and it became clear that we would need to rekindle their childhood memories of their own experiences of play and learning through our quarterly experiential workshops. Through posters setting out the routine for each play theme and, initially, direct support, many of the carers gained confidence and added their own themes, especially when they saw how much clients enjoyed these groups. Even those who were quite reserved saw the value of more directed play and were happy to follow one of the routines set out.

### Obstacle course

For the independently mobile clients who were typically overweight and unfit, it was important to introduce more physical activity which could be done in a group (Hung & Pang 2010; Park, Jeong & Bornman 2011). In addition to encouraging daily walks out of doors, it was a relatively easy task to set up a simple, accessible obstacle course. We just needed to find a dedicated space or at least space to store the obstacle course equipment so that it is easy to set up. We chose a few key items (a mat to sit on to sing a song at the beginning and end, a tunnel to climb through, roller to roll over, an area to throw and catch a ball, steps to go up and down, a trampoline to jump on and a balance beam to walk along). To be more inclusive, we also devised an ‘adapted’ version for groups of clients with more severe physical disabilities in wheelchairs and buggies. This included:

- The roller would be placed on each group member’s lap with their arms over the top (or as close to this as possible) and have their upper bodies moved gently forwards and backwards (to mobilise shoulders and trunk).
- The caregiver then facilitates each group member to hold a plastic soccer ball and facilitate gentle arm movement up/down/right/left and then throwing of the ball (mobilisation of arms and some sensory input).
- Then, the caregiver would take each group member in their buggy or wheelchair for a trip around the group involving a figure of eight, faster, slower, circles, or whatever the carer felt comfortable doing – preferably to some music.

#### Outcomes

It was quite simple to set up an obstacle course; it was not as easy to get groups of clients to go through the course in a set sequence, wait for their turn and offer encouragement to others who were taking their turn. Many of the clients were afraid of this new experience, while others wanted to rush in and do only the activities they enjoyed. Time played an important role as the groups slowly learnt what was expected of them when they came to do the obstacle course. Those individuals who were very afraid became more open to the new experiences and those who wanted to rush into it learnt to wait for their turn and, for short periods at least, remain on the mat. The group members became more aware of each other, whose turn it was, who was doing the activity correctly – or not. Once again there was more communication among the group members themselves and not just between the carer and each individual. There was lots of laughter (and groans and moans) and most important an opportunity to dispose of some physical energy that comes from being inactive. It also helped to modify challenging behaviour and the indoor activity was especially useful as it was not weather dependent. In addition, the physical endurance of many clients improved, getting up from the floor or going up steps became easier – all of which was useful for getting in and out of transport and for hospital visits and outings and thus facilitating general function and easing of care needs.

When first introducing the obstacle course for residents in buggies or wheelchairs, we found many of the group members were passive with several not enjoying having their bodies moved or having objects placed against them. Although not all group members came to enjoy this particular activity, it was beneficial to get some physical movement other than being placed in their seating devices, as well as getting some sensory stimulation from the roller/small ball. However, the majority of the group members loved the interaction with the carer and their peers as well as the opportunity to participate in a group and be in a different space.

### Individualised lap tray message for non-verbal clients

One of our observations was that visitors to centres were often overwhelmed by the clients with PIMD, not knowing how to interact with them. In worst-case scenarios, visitors were afraid of clients and so migrated towards interacting with the more physically able and communicative children and adults. We printed and pasted individually relevant, introductory messages on the tray tables of these residents facing away from the client, so that any visitor or new carer could quickly read and get to know something about the client. These individualised introductions included the resident’s name, what they liked or disliked and a suggestion or two on how to communicate with them through touch and voice. For example:

‘Hello, my name is …. I love being spoken to. If you touch my hand gently when you speak to me I can focus on you more easily’.

or:

‘Hello, my name is …. I love chatting to people. I can look up for “yes” and I blink for “no.” Please ask me *short* questions and *give me time to respond*!’

or:

‘Hello, my name is …. I am sleepy because of my medication. I smile when I like something and I close my eyes if I don’t. Hint: gently rub my hands or my back.’

#### Outcomes

The lap tray introductions made a big difference; visitors (and the carers themselves) were empowered to interact with the client with PIMD. Often the request for patience, a calm voice or a gentle touch in the lap tray message helped a visitor to see how the more severely physically disabled clients communicated their pleasure through a small smile or relaxed demeanour. For others, the visitor’s cued questions and comments about the client’s favourite soccer team or TV programme, and so on caused great excitement and enjoyment to both the client and the visitor. It meant that these clients with PIMD were included more when visitors came to the centres – and also reduced the stress levels of visitors. They could now easily access the name of the child/adult and had a few practical ideas of how they could start to interact with them. The lap tray introductions needed to be short and written in large font – otherwise new carers and visitors did not read them. We also found that covering the lap tray messages with clear adhesive contact protected them from getting wet or otherwise damaged – yet not too difficult to remove and replace when messages needed to be updated.

### Wristbands for drooling

We felt it was important that the clients who had drooling problems became more actively involved in solutions and so gave them the opportunity to use wristbands (such as those used by sports stars) made of absorbent material to wipe their own mouths. In order to avoid unrealistic expectations, three criteria were used to identify clients who could benefit from this programme, that is, they needed to be physically able to reach their mouths with their wrists as well as able to follow the simple instruction to lift their wrists to their mouths and only have a mild to moderate drooling problem (usually only using one bib per day).

#### Outcomes

It took some time to teach each client how and when to use the wristbands effectively, and then some time supporting carers in reminding these clients at regular intervals to swallow and wipe their mouths. After lots of repetition, most got the hang of it and all of a sudden no longer needed to wear bibs. Clients appeared more aware of their appearance and sat/walked more upright and loved the affirmation they got from carers when they remained dry. Clients started to indicate they wanted their wristbands put on when they got dressed. Many wristbands went missing initially and we learnt the importance of working with the laundry staff/carers/parents in the whole process so that they understood the idea and made sure the wristbands were valued along with the other laundry.

### Individualised seating

As seating of the more severely physically disabled clients improved with appropriate assistive devices at the centres, the therapy team realised that the independently mobile clients with intellectual disability did not always have a specific chair to sit on during mealtimes/small group stimulation time. There would be disagreements over who could sit on the available chairs and often clients sat on the floor or inappropriate chairs. This added to the difficulties of carers managing mealtimes/small group stimulation times as a lot of time was spent trying to gather together available tables and chairs. Along with management we devised a system that meant everyone could have their own chair with their photo on it. We hoped that these individualised chairs would make mealtimes and small group stimulation time calmer and more effective and would help modify some of the poor behaviour during meals.

#### Outcomes

This was another enormous learning curve for therapists and management staff on many different levels. There was always the challenge of finding enough suitable chairs, taking appropriate photographs of each of the clients and then finding a foolproof way to stick these photographs onto the chair. No sooner had we started a system than someone would find a way to take it off. It took a lot of time and perseverance from all staff to teach each of the clients about having their very own chair for mealtimes/small group stimulation time and that they should refrain from trying to pull/peel off the photographs. Eventually, it was decided to paste the photo on the chair and cover it with Perspex which was attached with screws to make it more difficult to remove. Duplicates of the photographs were made to play matching games during small group stimulation time to help clients learn about having their own chairs. As some of the clients became more aware of the fact that they now had their own chair they became protective of them and started indicating to others to sit on their own chair. There still remained a need to repair damaged ones on a regular basis, but it made mealtimes and small group stimulation time more ordered for the majority of clients and the carers had more time to facilitate those clients struggling with the concept of using their own chairs.

### Individual action plans

One of the difficulties faced when there are a large number of clients and different staff members involved is the difficulty of developing and implementing specific goals for individual clients within the programmes (Buntnix & Schalock 2010). We developed a word picture for each client noting communally decided goals in five different areas’ needs regarding (1) communication; (2) activities of daily living or sensory input; (3) movement or positioning; (4) socialisation and promoting good behaviour; and (5) knowing and learning (Appendix 2) that could be put up along with the client’s photograph and some information about likes and dislikes as well as photographs of their assistive devices.

#### Outcomes

Verbal feedback indicates that individual action plans do make it clearer to all levels of staff involved with the client as to what the goals are for individual clients. It also encourages visitors including parents to support the achievement of these goals. From a management perspective, it is useful in encouraging team discussions and is an easy reference as to what is expected of staff with regard to individual clients. The time involved in keeping the information updated regularly and the displayed document in good repair close to each client was noted as a challenge.

## Discussion and conclusion

The implementation of the simple ideas outlined above gave clients in the different settings more opportunities for learning in terms of daily activities and meaningful participation. This improved their physical functioning as well as individual communication strategies as they were challenged more on several different levels, in functional ways for daily-life situations in keeping with the ICF (WHO 2001). Furthermore, their carers, who were often surprised at how much clients understood and could do physically, were motivated to challenge them further. Clients no longer remained passive recipients of care but enjoyed more of (or even all) the outcomes described by Rosenbaum and Gorter including fitness, function, friendships, family factors and fun, that most important aspect of quality of life which is so often overlooked in planning programmes for persons with PIMD (2011:461).

Ongoing support, in the form of initial and repeated training and mentoring, of these simple ideas was a key factor in their success. This support focussed on all levels of staff (including cleaners, laundry staff and drivers) understanding the reason why the above systems were being introduced and what was required by each level of staff to make them sustainable. In addition to weekly visits, quarterly workshops focussed on experiential learning with discussions and questions leading to more formal input. These workshops were a wonderful opportunity for staff to reflect on their work within the context of the workshop’s theme, to discuss challenges and successes with specific clients and ‘to tell their stories’ that often led to group problem-solving and support (Van der Linde 2014). There were many challenges along the way that threatened the success of maintaining these simple programmes, in particular the idea that the carers felt they would add to an already heavy workload. Perseverance and knowing our goals had many rewards and feedback from the staff at the different centres indicated that support of the input/strategies devised by the staff themselves during the workshops was what affected change most (staff ‘buy in’). In some cases, there was a need to draw up timetables to clarify when individual staff would be involved in the different programmes. Some of the exciting ‘knock on’ effects of these simple ideas included the initiation of a prevocational group for adults at the residential centre, as well as employment of part-time therapists at the day care centres after we withdrew.

In conclusion, it was found that implementing an interdisciplinary daily routine was an effective way to introduce an equitable, effective and more holistic programme that could address the varying needs of individual clients with PIMD within day care and residential centres in low-resource contexts where the reality is often limited human resources. Long-term engagement of an interdisciplinary team (in this case, therapists and an educator) with carers to put in place doable, effective systems that do not add to an already heavy workload and can be sustainable over time without direct therapy support seems to show promise.

The simple ideas described in this article are flexible and can be used at home or in different centre-based contexts to encourage interaction, communication, movement and learning in clients with PIMD. We hope that sharing these ideas will allow carers and parents to identify what may be relevant for their specific environment, be it a day care or residential centre, or at home.

## Footnotes

1 ‘Buggy’ – this is the term used in the United Kingdom and in South Africa for a customised wheelchair which provides full body and head support for individuals who cannot sit independently and who need more support than what conventional wheelchairs offer (http://shonaquip.co.za/product/shona-buggy).

## APPENDIX 1: Monitoring and evaluation checklists.

### Checklist for small group stimulation time

Group assessed……………………………………………………….

Carer responsible……………………………………………………..

**Table:**

	Date								
1	Are the radio and television switched off?								
2	Are the group members in a circle?								
3	Is the group taking place in the correct venue?								
4	Does the group start with a greeting song?								
5	Is there a movement activity and facilitation for those who cannot do it themselves?								
6	Are the group members included in an activity that encourages them to use their hands in some way?								
7	Are the members not able to move themselves given a massage of their hands/feet?								
8	Are the members encouraged to communicate in some way with the carer running the group?								
9	Are the members encouraged to communicate in some way with other members of the group?								
10	Is there a picture/story book discussed with the group members?								
11	Does the carer use an appropriate tone and volume of voice (clear, calm, not too loud)?								
12	Does the group finish with a song?								
13	Does the carer check all items before replacing them in the appropriate toy box?								
14	Is the toy box suitably stocked?								
15	Has the small group used the sensory/play/obstacle course area in the past week?								
16	Has the weekly theme been incorporated?								

Concerns (if any):

**Table:**

Date	Area of concern	Action taken	Comment
			
			
			

### Checklist for equipment

Name of client………………………………………………………………………………………………………………………………

Date of birth…………………………………………………………………………………………………………………………………

Assistive device(s) used

1. …………………………………………………………………………………………………………………………………………………
2. …………………………………………………………………………………………………………………………………………………
3. …………………………………………………………………………………………………………………………………………………
4. …………………………………………………………………………………………………………………………………………………

Name of person responsible…………………………………………………………………………………………………………..

**Table:**

	Date								
1	Is the client using their device/s?								
2	**If using a buggy:**								
	Is the client’s name on their buggy?								
	Are the covers on the buggy and cushions?								
	Is the buggy clean?								
	Is the seat cushion right back?								
	Are there two side cushions?								
	Is there a sacral cushion?								
	Is there a head rest cushion?								
	Do the brakes work?								
	Are the wheels inflated?								
	Is the client’s bottom right back?								
	Does the client have their lap strap on?								
	Are the client’s feet positioned on their footplate?								
	Does the client have their tray table on? For non-verbal clients: do they have an up-to-date lap tray message?								
3	**If using a wheelchair:**								
	Is the client’s name on their wheelchair?								
	Is the wheelchair clean?								
	Does the wheelchair have its cushion on correctly?								
	Does the cushion have its cover on?								
	Are both footplates present?								
	Are both armrests present?								
	Is the client’s bottom right back?								
	Does the client have their lap strap on?								
	Are the client’s feet positioned on their footplates?								
	Does the client have their lap tray on?								
	Do the brakes work?								
	Are the wheels inflated?								
4	**If using a side lyer:**								
	Is the client’s name on their side lyer?								
	Is the side lyer clean?								
	Do the cushions of the side lyer have covers on?								
	Is the Velcro of the side lyer still working?								
	Is the client correctly positioned in the side lyer?								
5	**If using an orthopaedic splint: Hand/Foot**								
	Is the client wearing their splint/s?								
	Is the client’s name on their splint/s?								
	Is the splint/s clean?								
	Does the splint/s still fit?								
	Does the Velcro on the straps still work?								
	If the client is wearing a foot splint are they also wearing the correct size shoes with it?								
6	**Mobile clients:**								
	Does the client have his/her own chair to sit on?								
	Does the chair have the client’s name/photo on it?								
	Is the client sitting on it during stimulation time/meals?								
	Are they using the lap strap if this has been provided?								
7	**Other: for example, wristband, spectacles, hearing aid, medical bracelet**								
	Is the client wearing their wristband/spectacles/hearing aid/medical bracelet?								
	Is the wristband/spectacles/hearing aid/medical bracelet in good repair?								
	Is the wristband/spectacles/hearing aid/medical bracelet working well?								

Concerns (if any):

**Table:**

Date	Area of concern	Action taken	Comment
			
			
			

*Source*: Authors’ own work

## APPENDIX 2: Template for individual action plan word picture.

**Table:**

Name:	Age:	Individual Action Plan:	Date:
